# Antibacterial and Immunomodulatory Effects of *Limosilactobacillus reuteri* LMG P-27481 and Resveratrol Relevant for the Control of Upper Respiratory Tract Pathobionts

**DOI:** 10.3390/microorganisms14081771

**Published:** 2026-08-12

**Authors:** Roberto Mattioli, Daniel Di Risola, Francesca Sivori, Ornella Franzese, Ilaria Genovese, Paola Mastromarino, Luciana Mosca

**Affiliations:** 1 Department of Biochemical Sciences, Sapienza University of Roma, 00185 Rome, Italy; roberto.mattioli@uniroma4.it (R.M.); daniel.dirisola@uniroma1.it (D.D.R.); luciana.mosca@uniroma1.it (L.M.); 2 Laboratory of Biochemistry and Molecular Biology, Department of Movement, Human and Health Science, Università degli Studi di Roma “Foro Italico”, 00135 Rome, Italy; 3 Microbiology and Virology, San Gallicano Dermatological Institute, IRCCS, 00144 Rome, Italy; francesca.sivori@ifo.it; 4 Department of Systems Medicine, University of Rome Tor Vergata, 00133 Rome, Italy; franzese@uniroma2.it; 5 Departmental Faculty of Medicine, Unicamillus-Saint Camillus International University of Health Sciences, 00131 Rome, Italy; 6 Department of Public Health and Infectious Disease, Sapienza University of Roma, 00185 Rome, Italy; 7 Center for Research in Neurobiology Daniel Bovet, Sapienza University of Roma, 00185 Rome, Italy

**Keywords:** *Limosilactobacillus reuteri*, probiotics, resveratrol, upper respiratory tract infections

## Abstract

Although most upper respiratory tract infections (URTIs) have a viral etiology, antibiotics are widely prescribed, thus contributing substantially to antimicrobial resistance. This study investigated the potential beneficial effects of a novel strain, *Limosilactobacillus reuteri* LMG P-27481, against major URT pathobionts (*Staphylococcus aureus*, *Streptococcus pyogenes*, *Streptococcus pneumoniae*, *Moraxella catarrhalis*, and *Haemophilus influenzae*) with the aim of reducing or delaying antibiotic use. Antibacterial and immunomodulatory activities were evaluated using *L. reuteri* alone or combined with resveratrol, a natural compound with antiviral and anti-inflammatory properties. Both agents inhibited pathogen growth, while their combination showed additive antibacterial activity, particularly against Gram-negative bacteria. Antioxidant assays demonstrated significant antioxidant capacity for both agents, with enhanced effects in combination in NBT and ABTS assays but antagonistic in ORAC. Immune mediators (IL-6, IL-8, IL-1β, TNF-α, IL-25, IL-33, CCL17, CCL22) were assessed in epidermal, epithelial, and macrophage cell lines, together with epidermal integrity markers (Filaggrin, Loricrin, Involucrin) in a 3D reconstructed human skin model (EpiDermFT^TM^). *L. reuteri*, resveratrol, and their combination exerted cell-specific effects in the different models, modulating cytokine expression and production, and barrier integrity. Our findings support the potential efficacy of oral *L. reuteri* LMG P-27481 plus resveratrol in URTIs.

## 1. Introduction

Respiratory infections, particularly upper respiratory tract infections (URTIs), carry the highest disease burden globally and are the main cause of acute illnesses, especially impacting young children and leading to significant healthcare and societal costs [1]. The majority of upper respiratory tract infections (over 80%) are viral in origin, most commonly caused by rhinoviruses and coronaviruses. Furthermore, these infections can trigger secondary bacterial infections from pathogens that frequently colonize the upper respiratory tract of healthy individuals. While URTIs generally resolve on their own and antibiotics are ineffective against viral causes, these drugs remain heavily overprescribed [2,3] contrary to international guidelines [4], and have resulted in a concerning rise in antimicrobial resistance.

Moreover, the unnecessary use of broad-spectrum antibiotics can cause dysbiosis, i.e., a perturbation of resident microbiota with a reduction in the number of beneficial bacteria throughout the body. It is well known that a healthy microbiota is of paramount importance in maintaining a healthy lifestyle [5].

Both gut and respiratory microbiota play a crucial role in respiratory tract infections. Indeed, recent experimental and epidemiological evidence indicates an interaction between the intestinal microbiota and the lungs, called the gut–lung axis [6,7]. Mice lacking microbiota (i.e., germ-free) or those orally treated with antibiotics have impaired responses to viral or bacterial respiratory infections [8,9,10,11,12].

It has been shown that gut microbiota can support the antimicrobial defence of the respiratory tract through different mechanisms. Microbial metabolites produced by intestinal microorganisms (e.g., desaminotyrosine, short-chain fatty acids) are able to stimulate the lung’s production of type I interferons, with known antiviral activity [6,7,13]. Along with microbial metabolites, microbial membrane components derived from intact gut microbiota, including Toll-like receptor (TLR) ligands, also play a critical part in host defence against viral respiratory infections. Indeed, local and distal inoculation of Poly (I:C) (a TLR3 agonist) or peptidoglycan (a TLR2 agonist) rescued the immune impairment in antibiotic-treated mice [9].

In addition to the effect of gut microbiota, local microbes from the upper airway can act as a gatekeeper to the lower airways, providing resistance to colonization by respiratory pathogens [14]. Indeed, the respiratory microbiota plays a crucial role in maintaining ecological and immunological homeostasis within the respiratory tract [15,16].

Distinct microbiota profiles have been described in the respiratory tract of healthy infants starting from 1.5 months after birth [17], with progressive development in the nasopharyngeal microbiota occurring from infancy through early adulthood [18].

*Dolosigranulum* spp. and *Corynebacterium* spp. are essential commensals of the nasopharynx and are associated with lower rates of respiratory infections in infants [17,19,20] as well as with a reduced pneumococcal burden in children [20,21,22]. A similar protective role of *Prevotella* spp. against respiratory infection has been described, and an inverse relationship between *Prevotella* spp. and *Moraxella catarrhalis* has been reported in infants [23]. Respiratory microbiota can inhibit respiratory pathogens through competitive interactions or host crosstalk. Extracted secreted metabolites from *P. oris* inhibited *M. catarrhalis* in vitro, suggesting direct microbial competition, whereas no inhibition of *Streptococcus pneumoniae* was observed [23]. Instead, *P. melanogenica* was shown to enhance protection towards *S. pneumoniae* by inducing TNF-α and neutrophil activation, indicating modulation of the immune system [24]. The nasopharynx is a major reservoir of respiratory pathogens, and these pathogens are kept in check by local microbiota that can, however, be altered by antibiotics or viral infections [14,15,20,25].

Despite the widely documented capacity of probiotics to counteract bacterial and viral infections, few studies have been done on the identification of protective characteristics of probiotic strains for use in URT infections [26,27,28,29].

This study aimed to investigate the potential beneficial effect of a novel strain of *Limosilactobacillus reuteri*, LMG P-27481, against upper respiratory tract pathogens. This strain was selected for its antiviral and antibacterial properties and its positive effects on the immune system in different in vitro models and in mice [30].

The antibacterial and immunomodulating activities have been studied using, respectively, the supernatant of *L. reuteri* (*L. reuteri* SCS) or inactivated *L. reuteri* (HI *L. reuteri*) alone or in combination with resveratrol, a natural polyphenolic molecule with known antiviral activity against various respiratory viruses and anti-inflammatory effects [31,32].

## 2. Materials and Methods

### 2.1. Bacterial Strains and Growth Conditions

*Limosilactobacillus reuteri* LMG P-27481 was recovered from cryopreserved stock and cultured on de Man, Rogosa and Sharpe (MRS) agar plates (Difco, BD, Milan, Italy) at 37 °C under anaerobic conditions. Colonies from agar plates were subsequently inoculated into MRS broth and incubated under the same conditions to obtain liquid cultures used for supernatant preparation.

*Staphylococcus aureus* NCTC 6571, *Streptococcus pyogenes* LMG 21599, *Streptococcus pneumoniae* DSM 20566, *Moraxella catarrhalis* DSM 9143, recovered from cryopreserved stocks, were cultured on Brain Heart Infusion (BHI) agar (Oxoid) at 37 °C under aerobic conditions prior to the Minimal Inhibitory Concentration (MIC) testing. *Haemophilus influenzae* DSM 4690 was cultured in Schaedler agar (Liofilchem, Roseto degli Abruzzi, Italy) supplemented with 0.1% sterile defibrinated horse blood under microaerophilic conditions.

### 2.2. Minimal Inhibitory Concentration Assay

Spent culture supernatant (SCS) was obtained from liquid cultures of *L. reuteri* LMG P-27481 by centrifugation followed by sterile filtration (0.22 µm). The pH of the supernatant was recorded before testing. The MIC of *L. reuteri* LMG P- 27481 SCS, either alone or in combination with resveratrol (98%, from *Polygonum cuspidatum*, Shanghai Novanat Co., Ltd. (Shanghai, China)), was determined using the broth microdilution method with two replicates.

SCS was serially diluted in the Schaedler broth medium to obtain final concentrations ranging from 100% to 0.19% (*v*/*v*). The tested concentrations were 100, 50, 25, 12.5, 6.25, 3.12, 1.56, 0.78, 0.39, and 0.19% (*v*/*v*). Resveratrol testing was performed according to the CLSI M07 guidelines [33]. A stock solution of resveratrol was prepared in dimethyl sulfoxide (DMSO) and subsequently diluted in broth culture medium. The concentrations tested ranged from 640 to 1.25 µg/mL.

Pathogenic strains were adjusted spectrophotometrically to OD_600_ = 0.08–0.10, corresponding approximately to 10^8^ cfu/mL, and subsequently diluted 1:100 in broth or in sterile supernatant to obtain10^6^ cfu/mL.

A 96-well plate was filled to achieve a total volume of 100 μL with a final concentration of 5.0 × 10^5^ cfu/mL of the pathogens and the different dilutions of *L. reuteri* SCS or resveratrol. The microplates were incubated under aerobic conditions for all strains except for *H. influenzae* incubated under microaerophilic conditions. Growth was monitored at OD_600_ after overnight incubation at 37 °C.

The antimicrobial activity of *L. reuteri* SCS was assessed at both native pH and pH adjusted to neutrality (pH 7.0). Control wells containing serial dilutions of DMSO, used to dissolve resveratrol, were included to verify that the solvent did not exert inhibitory effects on pathogen growth.

### 2.3. Biofilm Assay

Biofilm disaggregation and prevention assays were performed using the SCS of *L. reuteri* LMG P-27481. Based on the MIC results, SCS was tested at 50% (*v*/*v*), corresponding to the lowest concentration that showed antimicrobial activity against all pathogens included in the study. For the disaggregation assay, pathogenic strains were inoculated at an initial concentration of 1.0 × 10^5^ cfu/mL in flat-bottom 96-well plates and incubated for 24 h at 37 °C under appropriate culture conditions to allow for biofilm formation. Following incubation, spent medium was removed and SCS from *L. reuteri* LMG P-27481 was added, followed by a further 24 h incubation period. In the prevention assay, pathogenic strains were incubated with SCS for 48 h. Sterile non-inoculated medium and untreated pathogen cultures were included as negative and positive controls, respectively. All experimental conditions were tested in octuplicate. Following incubation, planktonic cells were removed by washing wells twice with PBS (pH 7.4). Biofilms were fixed at 65 °C for 1 h and stained with 0.1% crystal violet for 15 min at room temperature. Wells were then rinsed twice with distilled water and air-dried. Bound crystal violet was solubilized with 33% (*v*/*v*) glacial acetic acid, and biofilm biomass was quantified by measuring optical density at 570 nm [34].

Statistical analysis was performed using one-way ANOVA followed by Dunnett’s multiple-comparison test versus the untreated control. Statistical significance was defined as *p* < 0.05 (*), *p* < 0.005 (**).

### 2.4. Heat Inactivation of L. reuteri

To identify optimal conditions for bacterial heat inactivation, two protocols were tested using 4.0 × 10^9^ cfu/mL and 4.0 × 10^7^ cfu/mL at 70 °C or 85 °C for 15 min. Comparison with untreated controls allowed for estimation of the reduction in viability.

### 2.5. Antioxidant Assays

#### 2.5.1. ABTS Radical Cation Scavenging Assay

A solution of 2,2′-azinobis-(3-ethylbenzothiazoline-6-sulfonic acid) radical cation (ABTS•^+^) was prepared in ethanol (EtOH) and adjusted to an absorbance of 0.7 at 734 nm.

Aliquots of 20 μL of bacterial samples, resveratrol, or ascorbic acid at different concentrations were added to 1 mL of the ABTS•^+^ solution. The reaction mixtures were incubated in the dark at room temperature for 6 min.

Following incubation and centrifugation, absorbance was measured at 734 nm using a spectrophotometer. A blank consisting of EtOH and the solvent used to dissolve the tested samples was used for baseline correction.

#### 2.5.2. NBT Reduction Assay

All reagents were freshly prepared immediately prior to use due to their susceptibility to degradation. The following solutions were used: 0.02 M Tris-HCl buffer (pH 8.0); 1 mM β-nicotinamide adenine dinucleotide, reduced form (β-NADH) in 0.05 M phosphate buffer (pH 7.4); 1 mM nitroblue tetrazolium (NBT) in bidistilled water; and 1 mM phenazine methosulfate (PM) in ethanol (EtOH).

The reaction mixture was prepared in a quartz cuvette by sequentially adding 838 μL of Tris-HCl buffer (0.02 M, pH 8.0), 20 μL of bacterial samples, resveratrol, or ascorbic acid at different concentrations, 50 μL of NBT solution (1 mM), 77 μL of β-NADH solution (1 mM), and 15 μL of PM solution (1 mM).

Absorbance was measured kinetically at 560 nm immediately after reagent addition and after 15 s. The change in absorbance (ΔAbs) was used to calculate the percentage inhibition relative to the control, consisting of ethanol and the solvent used to dissolve the tested samples.

### 2.6. Oxygen Radical Absorbance Capacity (ORAC) Assay

The ORAC assay was performed in 96-well microplates. Each well was prepared with 150 μL of phosphate buffer (75 mM, pH 7.0) containing 3.4 μg/mL R-phycoerythrin and 25 μL of bacterial samples, resveratrol, or Trolox (6-hydroxy-2,5,7,8-tetramethylchroman-2-carboxylic acid), used as a standard, at different concentrations.

The plate was incubated at 37 °C for 15 min. Subsequently, 25 μL of 2,2′-azobis(2-amidinopropane) dihydrochloride (AAPH, 128 mM) was added to each well to achieve a final concentration of 16 mM.

Fluorescence intensity was measured every 5 min for a total of 120 min using a microplate reader, with excitation and emission filters set at 492/10 nm and 570/10 nm, respectively. Results were calculated based on the differences in the areas under the fluorescence decay curves (AUC), obtained by plotting fluorescence intensity versus time.

Blanks were prepared using the same reaction mixtures described above, without the addition of AAPH.

### 2.7. Immortalized Cells and Ex Vivo Tissues Growth Conditions

Caco-2, HaCaT, and RAW264.7 cell lines were maintained at 37 °C in a humidified atmosphere containing 5% CO_2_. Caco-2 cells were cultured in Dulbecco’s Modified Eagle Medium (DMEM), while HaCaT and RAW264.7 cells were maintained in high-glucose DMEM. All culture media were supplemented with 10% fetal bovine serum (FBS), 1% penicillin–streptomycin, and 2 mM of glutamine. For treatment experiments, Caco-2, HaCaT, and RAW264.7 cells were seeded in 6-well plates at densities of 2.0 × 10^5^, 5.0 × 10^5^, and 3.0 × 10^5^ cells per well, respectively. After 24 h, cells were subjected to the respective experimental treatments. Caco-2 cells were stimulated with phorbol 12-myristate 13-acetate (PMA, 20 ng/mL) and ionomycin (1 μg/mL), followed by treatment with bacteria and resveratrol (10 μM) for 6 h. HaCaT cells were stimulated with interferon-γ (IFN-γ, 20 ng/mL) and subsequently treated with bacteria and resveratrol (3 μM) for 6 h. RAW264.7 cells were stimulated with lipopolysaccharide (LPS, 100 ng/mL) and treated with bacteria and resveratrol (3 μM) for 6 h.

Three-dimensional full-thickness reconstructed human skin tissues (EpiDermFT™, EFT-400; MatTek Corporation, Ashland, MA, USA) were equilibrated and maintained in the proprietary serum-free maintenance medium supplied by the manufacturer at 37 °C in a humidified atmosphere containing 5% CO_2_, according to the manufacturer’s instructions.

Three-dimensional full-thickness reconstructed human skin tissues were stimulated with interferon-γ (IFN-γ, 20 ng/mL) and subsequently treated with bacteria and resveratrol (3 μM) for 6 and 24 h.

### 2.8. Enzyme-Linked Immunosorbent Assay (ELISA)

Before the analysis of cytokine production, the optimal non-toxic concentrations of resveratrol and bacteria for each cell line were determined through MTT and crystal violet assays. Prior to staining, cells were gently washed several times with phosphate-buffered saline (PBS) to remove residual medium, cellular debris, and non-viable cells. For the staining procedure, 50 µL of staining solution was added to each well as follows: MTT solution (5 mg/mL in PBS) for metabolic activity assessment, or crystal violet solution (2.5 mg/mL in 20% methanol) for biomass quantification. Plates treated with MTT were incubated at 37 °C for 2 h, whereas plates stained with crystal violet were incubated for 20 min under gentle agitation on a rocking platform. Following incubation, wells were washed with PBS to remove excess dye. The retained dye was subsequently solubilized using dimethyl sulfoxide (DMSO) in order to obtain a homogeneous solution suitable for spectrophotometric analysis. Absorbance was measured for each well at 570 nm using a microplate reader. The obtained values were normalized to the untreated control condition.

Culture media of the three cell lines were analysed for IL-6, IL-8, TNF-α and IL-1β using specific anti-human (for Caco-2 and HaCaT cells) and anti-mouse (for Raw264.7 cells) ELISA kits (4A Biotech, Beijing, China) following the manufacturer’s instructions. All the experiments were performed in duplicate.

### 2.9. Cytokine Gene Expression in HaCaT Epithelial Cells

Total RNA was extracted from HaCaT cells with TRIZOL reagent (Invitrogen™ bought from Thermo Fisher Scientific, Waltham, MA, USA) after 6 h of incubation with samples to be tested, following the manufacturer’s protocol.

An amount of 4 μg of total RNA was reverse transcribed to cDNA by GoTaq 2 Step RT qPCR System kit (Promega, Madison, WI, USA) according to the manufacturer’s instructions. cDNA was tested for gene expression with specific PCR primer sets for *IL-25*, *IL-33*, *CCL17*, *CCL22* (H_IL25_For: GCTGCTCTACCACAACCAGACT; H_IL25_Rev: GCACACACACACAAGCTAAGGA; H_IL33_For: CCATTACTTTTGCTTTGGAGGA; H_IL33_Rev: TCATTTGAGGGGTGTTGAGACT; H_CCL17_For: GGGAGTGCTGCCTGGAGTA; H_CCL17_Rev: TGCACAGTTACAAAAACGATGG; H_CCL22_For: ACTGCACTCCTGGTTGTCCTC; H_CCL22_Rev: GCAGACGGTAACGGACGTAAT—Tm: 58 °C) and run on an CFX96 BioRad thermocycler (Bio-Rad Laboratories, Hercules, CA, USA). Quantitative real-time PCR results were normalized to *RPS27a* (H_RPS27a_For: CCTTACGGGGAAGACCATCA; H_RPS27a_Rev: TGCCAGCAAAGATCAGTCTCT—Tm: 58 °C) expression (housekeeping gene). Fold change in gene expression was expressed as 2^−ΔΔCt^.

### 2.10. Filaggrin, Loricrin and Involucrin Gene Expression in EpiDermFT^TM^

Total RNA was extracted from EpiDermFT^TM^ tissues with TRIZOL reagent (Invitrogen™ bought from Thermo Fisher Scientific) after 6 and 24 h of incubation with samples to be tested, following the manufacturer’s protocol.

An amount of 4 μg of total RNA was reverse-transcribed to cDNA using the GoTaq 2 Step RT qPCR System kit (Promega) according to the manufacturer’s instructions.

cDNA was tested for gene expression with specific PCR primer sets for *IL-25*, *IL-33*, *IL-37*, *FLV*, *LOR*, *INV* (H_IL37_For: GCTGCCCAAAAGGAATCAG; H_IL37_Rev: GTCACCCCAACAGGCTCA; H_FLG_For: CTGAAAGGCAAAAAGGTCAATC; H_FLG_Rev: TTCCGTGCTGAGAGTGTCTAAA; H_LOR_For: GCGAAGGAGTTGGAGGTGTT; H_LOR_Rev: TGGGGTTGGGAGGTAGTTGT; H_INV_For: TTCCTCCTCCAGTCAATACCC; H_INV_Rev: CATTCTTGCTCAGGCAGTCC—Tm: 58 °C) and run on an CFX96 BioRad thermocycler. Quantitative real-time PCR results were normalized to *RPS27a* expression (housekeeping gene). Fold change in gene expression was expressed as 2^−ΔΔCt^.

### 2.11. Statistical Analysis

The software programs EXCEL^®^ and GraphPad Prism 6.0 were used to analyse and plot the data. All the results are presented as mean ± standard deviation (SD). The analysis of statistical significance was performed using one-way ANOVA. The level of significance was set at *p* < 0.05.

## 3. Results

### 3.1. Antibacterial Activity of L. reuteri LMG P-27481 Spent Culture Supernatant and Resveratrol

The activity of the secreted metabolites of *L. reuteri* LMG P-27481was explored by testing the antimicrobial activity of SCS in MIC assays. SCS obtained after overnight culture of *L. reuteri* (final concentration 1.4 × 10^9^ cfu/mL) was tested at the original pH value (4.4) and at neutral pH.

The MIC values, reported in Table 1, demonstrate that the growth of all potential pathogens is at least partially inhibited by metabolites produced by the *Lactobacillus* strain.

When the pH of SCS was brought to neutral values, the inhibition of Gram-positive pathogens disappeared (*S. aureus*) or was reduced approximately 4-fold, suggesting that acidic compounds were responsible for the inhibition. Conversely, no change in inhibitory activity against Gram-negative bacteria (*M. catarrhalis*, *H. influenzae*) was observed. It is important to underline that the pH of the medium in the wells corresponding to the MICs of these bacteria was 5.8 and 6.3, respectively; furthermore, the growth of *M. catarrhalis* was not inhibited in BHI brought to pH 4.5.

Resveratrol produced growth inhibition of the test pathogens at similar MIC values (80–160 µg/mL). DMSO, used to prepare a stock solution of resveratrol, was devoid of any inhibitory effect (MIC > 5%) at the amount present in the maximal concentration tested (DMSO 4%, resveratrol 640 µg/mL).

The tested pathogens are frequently present in the upper respiratory tract of healthy individuals (pathobionts) and can cause disease following viral infections in this region. Because resveratrol has demonstrated activity against many respiratory viruses [31], we evaluated the combined effect of *L. reuteri* SCS and resveratrol to assess the potential use of this combination in upper respiratory tract infections. In these trials, the highest concentration of resveratrol that did not exhibit an inhibitory effect against each of the bacteria studied was used (40 µg/mL), in order to exclude that resveratrol per se could exert the inhibitory effect and to maximise the potential additive effect of resveratrol and *L. reuteri* SCS.

As reported in Table 2, a combination of *L. reuteri* SCS at the native pH and resveratrol was strongly effective against Gram-negative bacteria. A 4-fold reduction in MIC values compared with those observed with SCS alone was found, indicating a significant additive action against *M. catarrhalis* and *H. influenzae*. On the contrary, no effect of resveratrol on the MIC values of *L. reuteri* SCS against Gram-positive pathogens was observed.

The results obtained with the combination of *L. reuteri* SCS at the neutral pH and resveratrol seem to confirm that the acidity provides a significant contribution to the observed antimicrobial action of *L. reuteri* LMG P-27481 metabolites against Gram-positive pathogens. In fact, the neutralization of SCS fostered pathogen growth even in the presence of resveratrol.

The neutralisation of SCS did not significantly affect the antimicrobial action of the SCS-resveratrol combination towards Gram-negative bacteria, since the MIC values only changed 1-fold compared to the acid supernatant.

### 3.2. Biofilm Disaggregation and Prevention Activity of L. reuteri LMG P-27481 SCS

**Figure 1:**
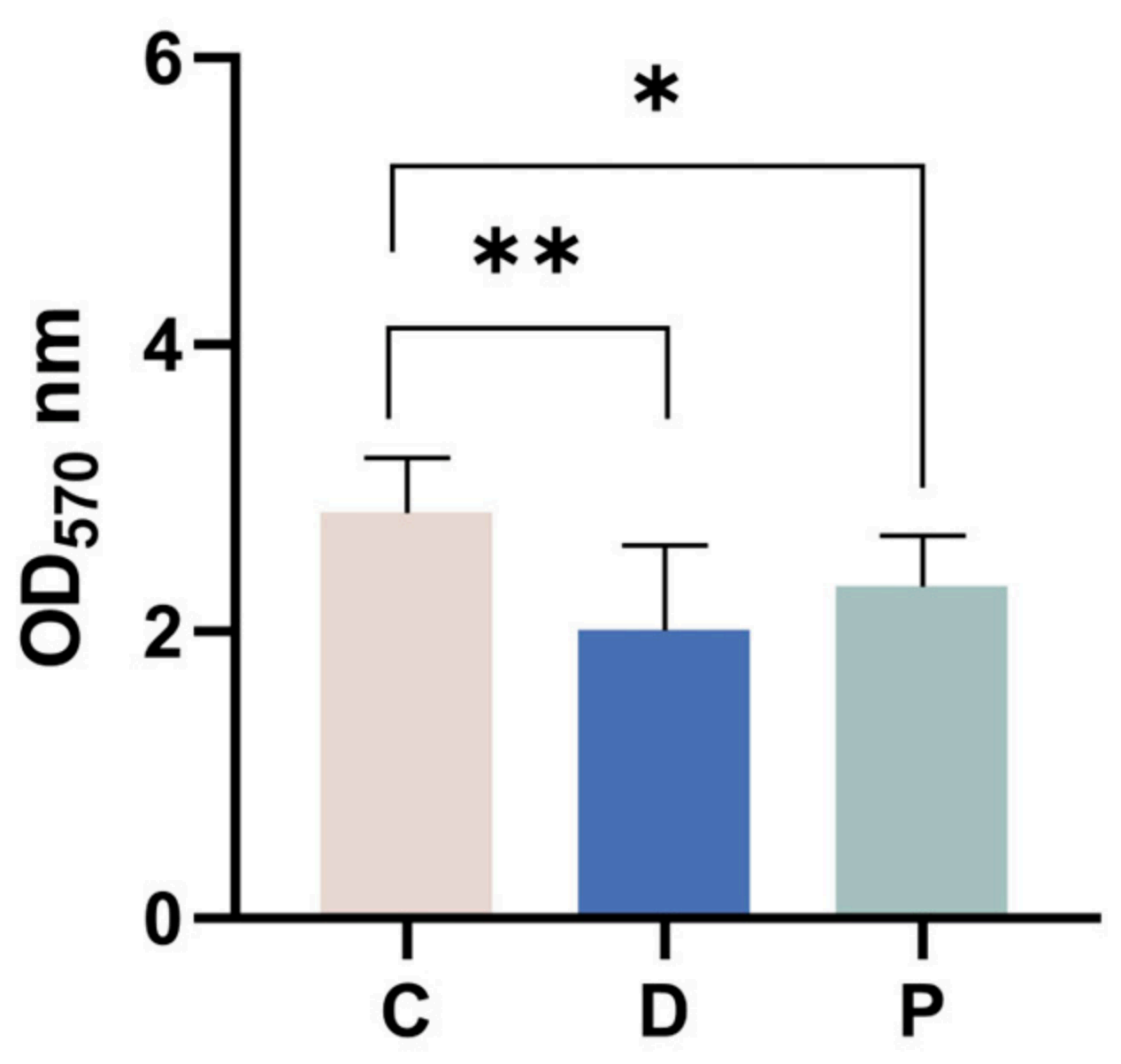
Effect of *L. reuteri* SCS on *S. pyogenes* LMG 21599 biofilm. Biofilm biomass was quantified by crystal violet staining and measured as optical density at 570 nm (OD570). Results are expressed as mean ± standard deviation of eight replicates. Statistical significance was assessed by one-way ANOVA followed by Dunnett’s multiple comparisons test; * *p* < 0.05, ** *p* < 0.005 vs. untreated control. C: Control; D: Disaggregation; P: Prevention.

The potential of *L. reuteri* LMG P-27481 SCS to interfere with biofilm dynamics was investigated across all pathogens included in the study, testing its ability to both disrupt pre-formed biofilms and prevent their formation. Among these, only *S. pyogenes* LMG 21599 showed statistically significant differences compared to the untreated control; data from the remaining strains are therefore not shown. As shown in Figure 1, treatment with SCS resulted in a statistically significant reduction in biofilm biomass, both when applied to pre-formed biofilms (disaggregation assay; *p* < 0.005) and when co-incubated with the pathogen from the start of the experiment (prevention assay; *p* < 0.05). These findings indicate that SCS from *L. reuteri* LMG P-27481 exerts a significant inhibitory effect on both biofilm formation and pre-formed biofilms of *S. pyogenes*.

### 3.3. In Vitro Antioxidant Activity of Resveratrol, Heat-Inactivated L. reuteri and Their Combination

The use of inactivated bacteria represents a promising strategy compared with the administration of live microorganisms, particularly in pharmaceutical and dermatological applications. Inactivated bacterial preparations retain several bioactive components, including structural molecules and bacterial-derived factors capable of modulating host responses, while avoiding the potential risks associated with the presence of viable microorganisms. Unlike live bacterial formulations, whose viability and functional properties may be affected by environmental conditions, inactivated preparations provide more consistent and reproducible biological effects.

Therefore, in the antioxidant assays, heat-inactivated *L. reuteri* was used. In preliminary tests, we verified the optimal conditions required to achieve the inactivation of different bacterial amounts. While treatment at 70 °C did not completely inactivate bacteria at either concentration, treatment at 85 °C resulted in complete inactivation. No colonies were observed on any plate, regardless of the bacterial concentration tested. Therefore, heat inactivation at 85 °C for 15 min was selected as the optimal protocol and was used in antioxidant and immunomodulating assays.

All in vitro antioxidant assays were performed at different bacterial concentrations, and the activity curves were compared with those of known antioxidant molecules, such as ascorbic acid, resveratrol, and Trolox. In addition, the bacterial concentrations showing the highest activity were tested in combination with resveratrol to assess potential synergistic, additive, neutral, or inhibitory effects.

To this end, mixtures of heat-inactivated bacteria were subjected to eight serial dilutions: 4.0 × 10^9^, 1.0 × 10^9^, 3.3 × 10^8^, 1.1 × 10^8^, 3.7 × 10^7^, 1.2 × 10^7^, 4.1 × 10^6^ and 1.4 × 10^6^ cfu/mL. Twenty µl of each dilution was then used in all antioxidant assays following the respective protocols.

#### 3.3.1. NBT Assay

In the NBT assay, which measures the ability to scavenge the superoxide radical (•O_2_^−^), resveratrol was the most effective compound, with an IC_50_ of 0.090 ± 0.012 mM, significantly lower than that of ascorbic acid (0.665 ± 0.026 mM).

Bacteria also exhibited strong scavenging activity against the superoxide radical, reaching inhibition values close to 100% at the highest concentrations tested, with an IC_50_ of 2.6 × 10^7^ ± 1.5 × 10^6^ cfu/mL. Moreover, the combination of resveratrol and bacteria reduced the resveratrol IC_50_ to 0.006 ± 0.002 mM, suggesting an additive antioxidant effect (Table 3).

**Figure 2:**
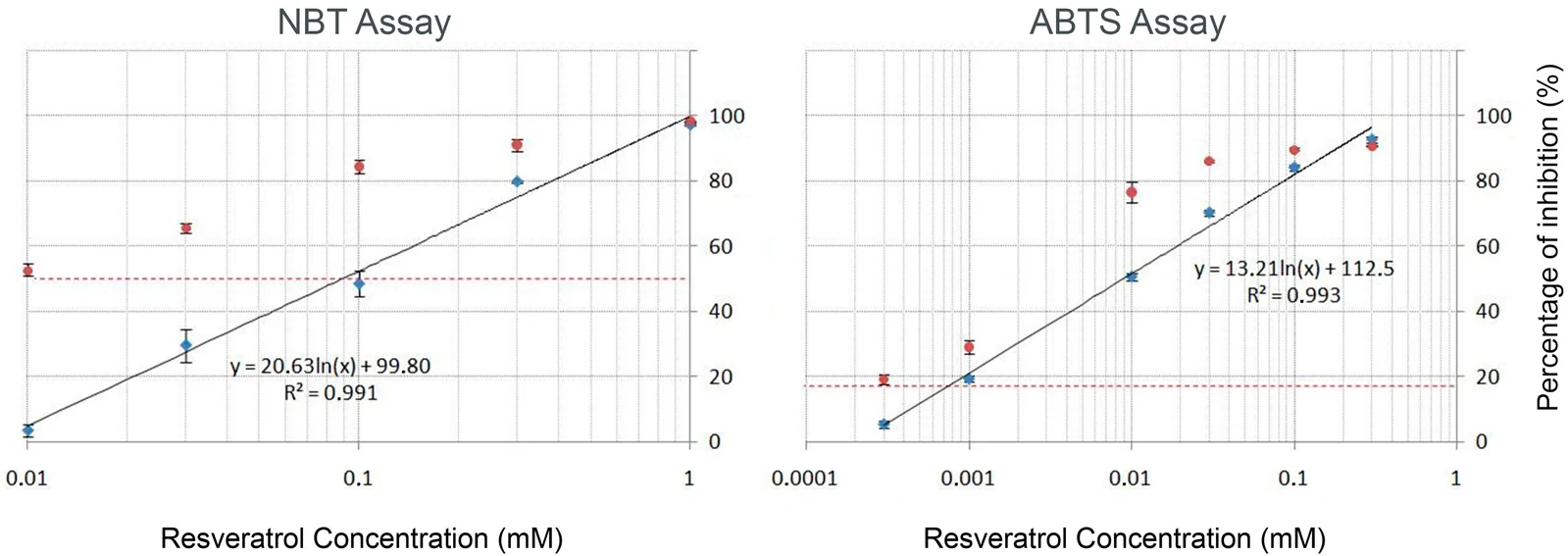
NBT and ABTS assays of resveratrol, HI *L. reuteri* and their combination. Trend of antioxidant activity (expressed as percentage), measured by the NBT (**left**) and ABTS (**right**) assays, as a function of resveratrol concentration. Blue: resveratrol; red: combination of resveratrol and bacteria at a concentration of 2.0 × 10^7^ cfu/mL. The dotted red line indicates the antioxidant activity for bacteria alone.

As the concentration of resveratrol in the mixture decreased, the percentage inhibition returned to levels comparable to those observed for bacteria alone (Figure 2).

The antioxidant capacity exhibited by bacteria in the NBT assay, both alone and in combination with resveratrol, is not unexpected, as bacterial proteins and enzymatic machinery can detoxify reactive oxygen species such as the superoxide radical. However, these results suggest that the heat inactivation protocol, while abolishing bacterial viability, did not completely impair the activity of antioxidant molecules.

#### 3.3.2. ABTS Assay

In the ABTS assay, which evaluates scavenging activity against the ABTS•^+^ radical by monitoring the decrease in absorbance at 734 nm after a fixed incubation time, both ascorbic acid and resveratrol showed strong activity, with IC_50_ values of 0.007 ± 2.39 × 10^−5^ mM and 0.010 ± 0.004 mM, respectively.

It was not possible to calculate the IC_50_ for bacteria, as the highest tested concentration yielded only 16.90 ± 0.38% inhibition. However, the combination of resveratrol and bacteria resulted in a lower IC_50_ (0.003 ± 0.0007 mM) compared to resveratrol alone, suggesting an additive antioxidant effect similar to that observed in the NBT assay (Table 3). As the resveratrol concentration decreased, inhibition values returned to levels comparable to those observed for bacteria alone (Figure 2).

#### 3.3.3. ORAC Assay

Finally, the ORAC assay, which determines the ability of a compound to inhibit oxidative degradation induced by peroxyl radicals (R–O–O•) using a fluorescent probe, revealed that resveratrol exhibited good inhibitory activity at 10 µM, corresponding to 27.397 ± 0.503 mM Trolox equivalents.

**Figure 3:**
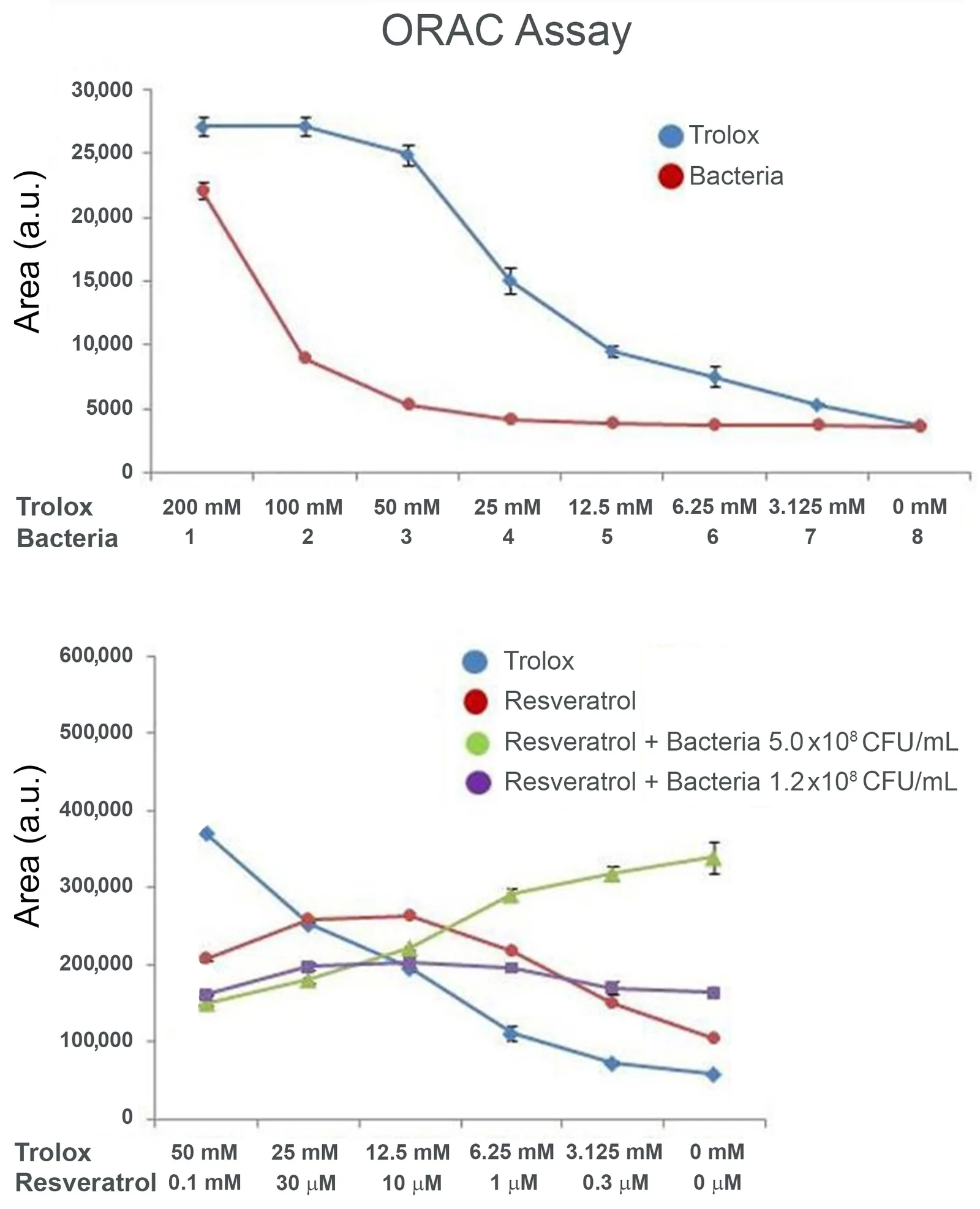
ORAC assay of resveratrol, *L. reuteri* and their combination. Trend of antioxidant activity measured by the ORAC assay. (**Upper panel**) Activity measured as a function of Trolox concentration (range 200 mM–0 mM; blue line) and bacteria (red line; numbers 1–8 correspond to bacterial concentrations as follows: (1) 5.0 × 10^8^; (2) 1.2 × 10^8^; (3) 4.2 × 10^7^; (4) 1.4 × 10^7^; (5) 4.6 × 10^6^; (6) 1.5 × 10^6^; (7) 5.1 × 10^5^; (8) 1.7 × 10^5^ cfu/mL). (**Lower panel**) Activity measured as a function of Trolox concentration (blue line), resveratrol (red line), and combinations of resveratrol with two different bacterial concentrations (5.0 × 10^8^ cfu/mL, green line; and 1.2 × 10^8^ cfu/mL, purple line) identified as effective in preliminary experiments reported in the upper panel.

Bacteria at a concentration of 5.0 × 10^8^ cfu/mL also showed strong inhibitory capacity (42.753 ± 1.642 mM Trolox equivalents), which decreased sharply with decreasing concentration (Figure 3). However, the combination of 10 µM resveratrol and 5.0 × 10^8^ cfu/mL bacteria reduced the inhibitory capacity to 18.269 ± 0.461 mM Trolox equivalents (Table 3). As the concentration of resveratrol decreased, the inhibition value of the mixture, expressed as mM Trolox equivalents, decreased as well (Figure 3).

Although the different assays yielded non-uniform responses and the in vitro antioxidant activity of resveratrol, heat-inactivated *L. reuteri*, and their combinations appeared to be assay-dependent, these results highlight a notable antioxidant capacity of the bacteria, both alone and in combination with resveratrol, particularly against the superoxide radical (•O_2_^−^), a reactive oxygen species produced by cells under stress or injury conditions.

### 3.4. Cytokine Production in Caco-2, HaCaT, and Raw264.7 Cells Treated with Resveratrol, HI L. reuteri, and Their Combination

In order to evaluate the immunological effects of this combination on epithelial and mucosal surfaces, we utilized three different well-characterized cell lines. Caco-2 cells were selected as mucosal intestinal cells, HaCaT cells as representative of anterior nares epithelial cells which is the initial site of interaction of respiratory microorganisms, and RAW264.7 murine macrophages as key cells of the immune system involved in mediating antibacterial systemic responses.

In these assays heat-inactivated *L. reuteri* cells were used in the different cell lines in order to evaluate the contribution of surface PAMPs (Pathogen-Associated Molecular Patterns) in host defence against infections.

After determining, through MTT and crystal violet assays, the optimal non-toxic concentrations of resveratrol and bacteria for each cell line, Caco-2 cells were stimulated with ionomycin and phorbol myristate acetate (PMA), HaCaT cells with IFN-γ, and Raw264.7 cells with lipopolysaccharide (LPS). Cytokine production was then evaluated by ELISA to assess changes following treatment with resveratrol, HI *L. reuteri*, and their combination.

**Figure 4:**
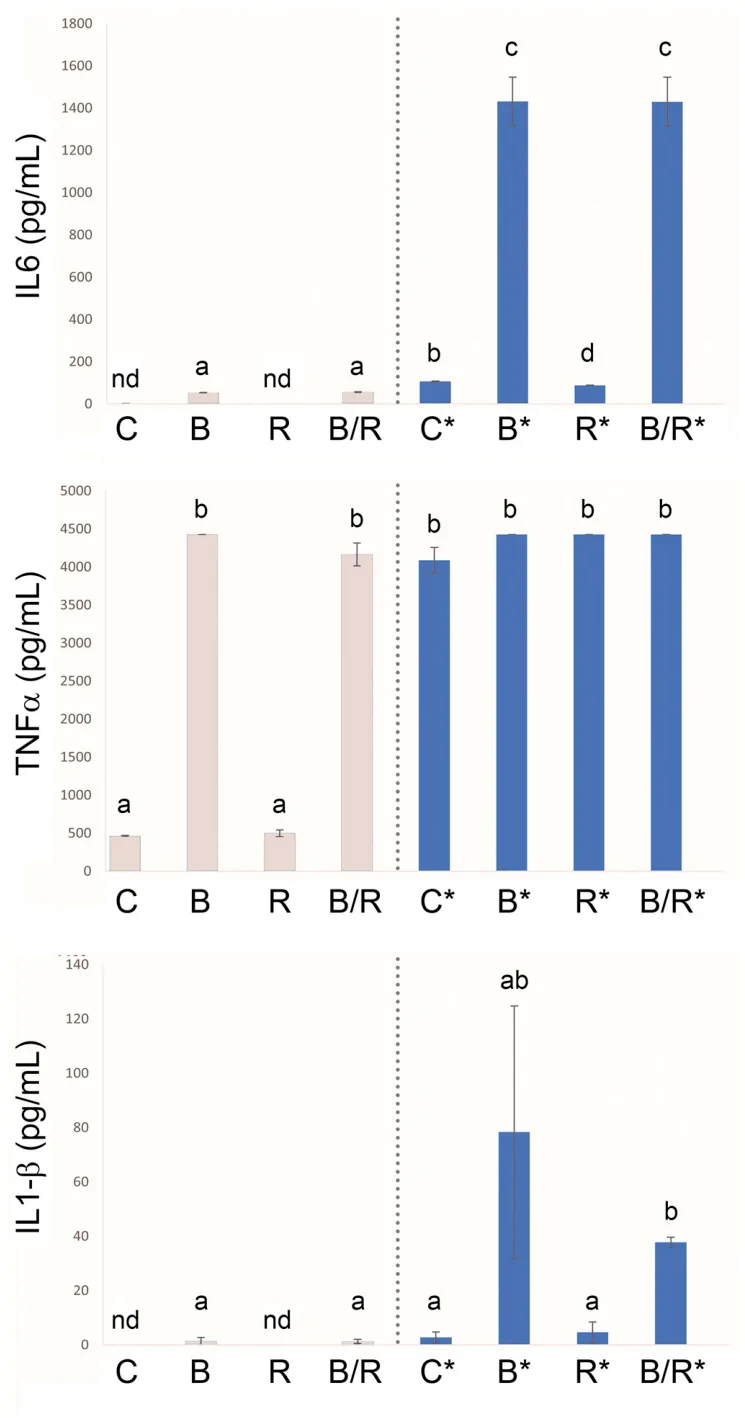
Cytokine production in RAW 264.7 cells. Cytokine production in RAW 264.7 cells, unstimulated and stimulated with LPS at a final concentration of 100 ng/mL. Data are presented as the mean of two independent biological replicates ± standard deviation. Letters above each bar indicate statistically different groups, as determined by multiple comparisons, with *p*-values < 0.05. *: stimulation with LPS; C: Control; B: Bacteria, final concentration 0.5 × 10^8^; R: Resveratrol, final concentration 3 µM; B/R: Bacteria + Resveratrol. nd: not detected.

In untreated Raw264.7 murine macrophages (no LPS), administration of HI *L. reuteri* alone or in combination with resveratrol resulted in a statistically significant increase in IL-6, TNF-α, and IL-1β levels in the culture medium. Furthermore, treatment with HI *L. reuteri* alone or in combination with resveratrol in LPS-activated Raw264.7 cells led to a significant increase in IL-6 and IL-1β levels compared to relative controls (Figure 4). HI *L. reuteri* induced significantly higher levels of IL-6 after stimulation compared to untreated and LPS-stimulated cells.

**Figure 5:**
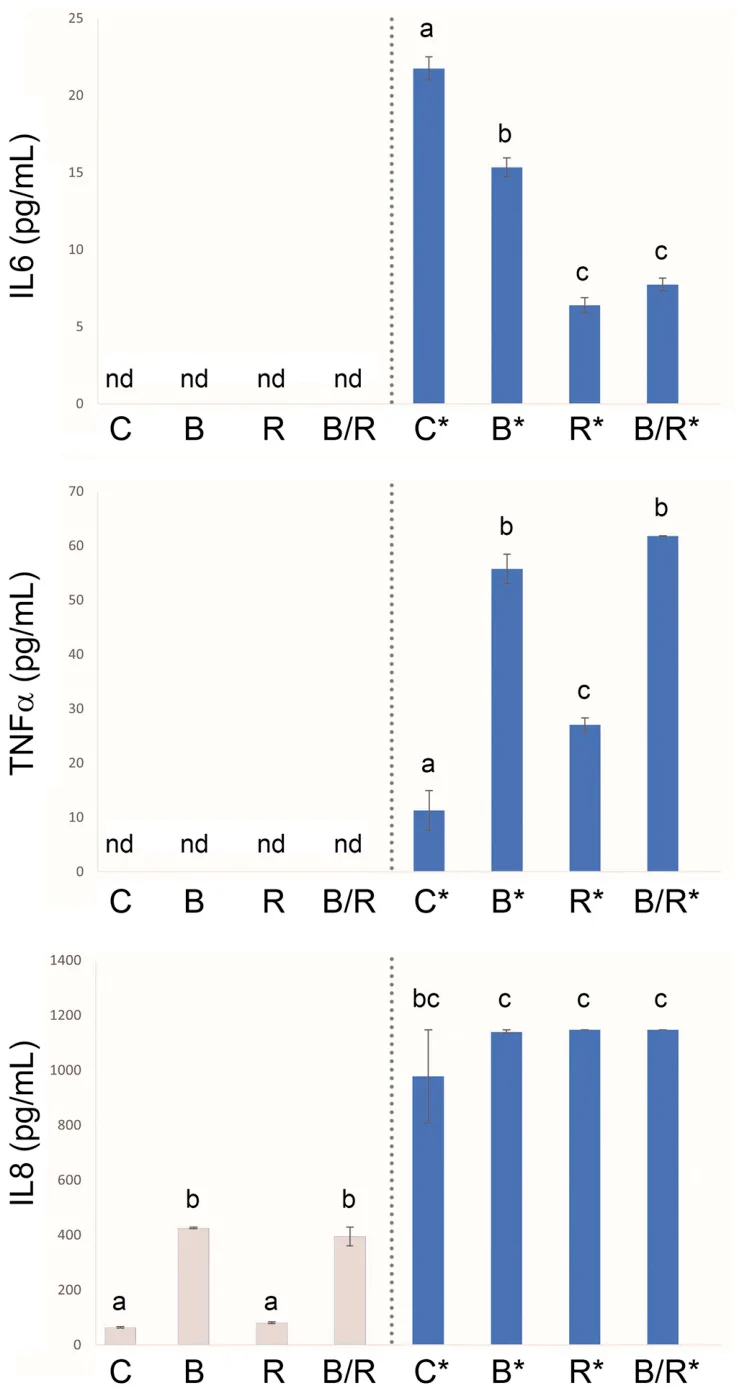
Cytokine production in Caco-2 cells. Cytokine production in Caco-2 cells, unstimulated and stimulated with PMA at a final concentration of 20 ng/mL and ionomycin at a final concentration of 1 µg/mL. Data are presented as the mean of two independent biological replicates ± standard deviation. The letters above each bar indicate statistically different groups, as determined by multiple comparisons, with *p*-values < 0.05. *: stimulation with PMA and ionomycin; C: Control; B: Bacteria, final concentration 0.2 × 10^9^; R: Resveratrol, final concentration 10 µM; B/R: Bacteria + Resveratrol. nd: not detected.

Unstimulated human intestinal Caco-2 cells showed no cytokine production except for low levels of IL-8, which were significantly increased by the presence of HI *L. reuteri* alone or in combination with resveratrol. In activated cells, treatment with resveratrol, HI *L. reuteri*, and their combination produced a complex pattern. IL-6 levels were markedly reduced following all treatments compared to the relative control. In contrast, IL-8 levels remained high and comparable to the control, indicating no modulation. Treatment with HI *L. reuteri*, resveratrol, and their combination enhanced TNF-α production, with a more pronounced effect observed for bacteria alone or in combination with resveratrol than for resveratrol alone (Figure 5). Moreover, HI *L. reuteri* induced significantly higher levels of TNF-α after stimulation compared to untreated and stimulated cells.

**Figure 6:**
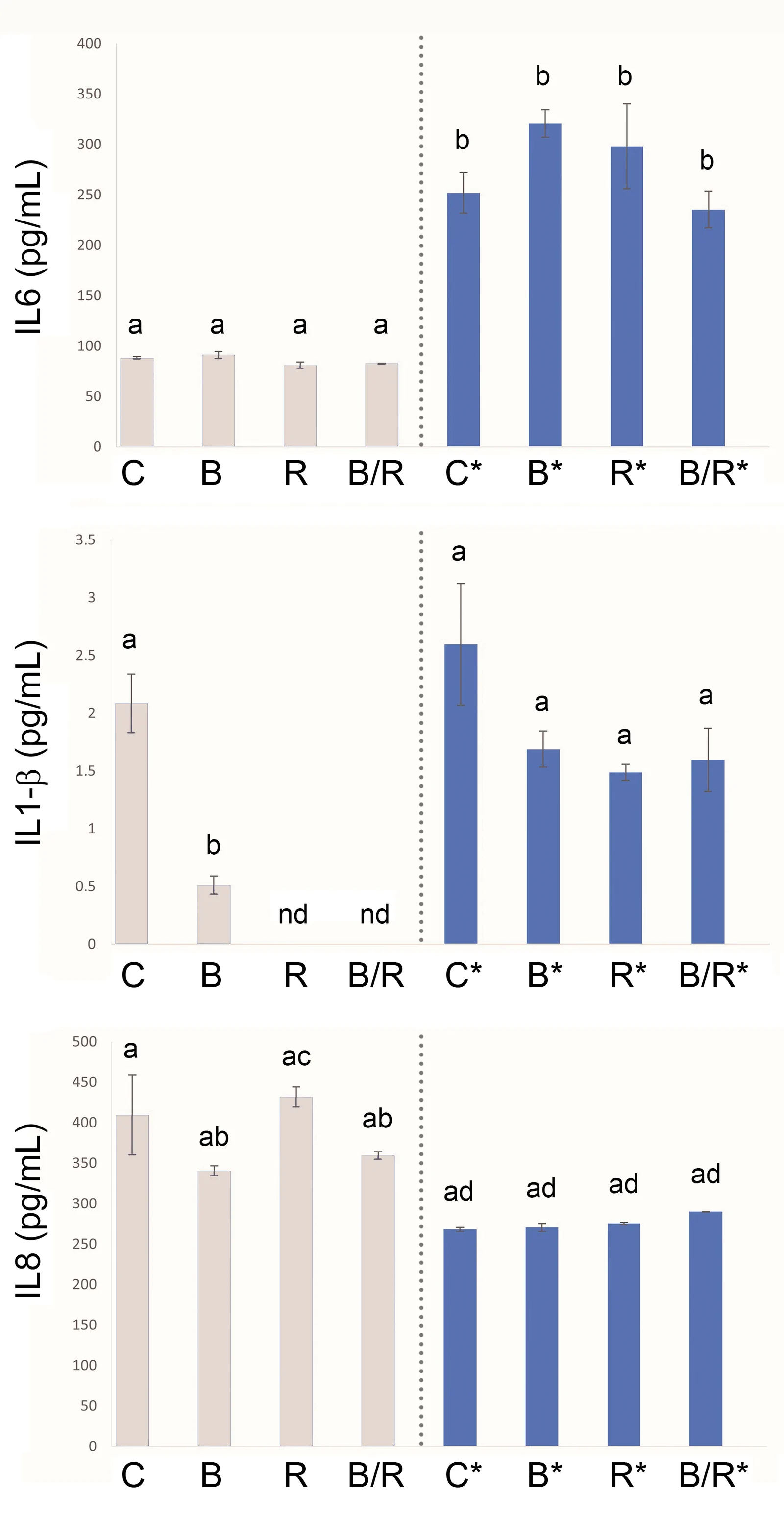
Cytokine production in HaCaT cells. Cytokine production in HaCaT cells, stimulated and unstimulated with IFN-γ at a final concentration of 20 ng/mL. Data are presented as the mean of two independent biological replicates ± standard deviation. Letters above each bar indicate statistically different groups, as determined by multiple comparisons, with *p*-values < 0.05. *: stimulation with IFN-γ; C: Control; B: Bacteria, final concentration 0.2 × 10^9^; R: Resveratrol, final concentration 3 µM; B/R: Bacteria + Resveratrol. nd: not detected.

In human non-activated HaCaT keratinocytes, bacteria, resveratrol and their combination did not influence IL-6 and IL-8 production, while significantly reducing IL-1β, with resveratrol being the most effective (Figure 6). Surprisingly, IFN-γ stimulation induced a significant increase in IL-6 production only, while IL-1β and IL-8 production were not altered compared to non-activated cells. Treatment with HI *L. reuteri*, resveratrol, and their combination did not affect IL-6, IL-1β, or IL-8 levels in activated cells.

### 3.5. Cytokine Gene Expression

The gene expressions of IL-25, a canonical type 2 response-inducing cytokine, IL-33, an alarmin cytokine involved in viral infection, as well as in allergic and non-allergic airway inflammation and asthma, and CCL17 and CCL22, chemokines that play key roles in regulating the immune response and inflammation [35,36], were analysed by real-time PCR at 6 h after treatment.

**Figure 7:**
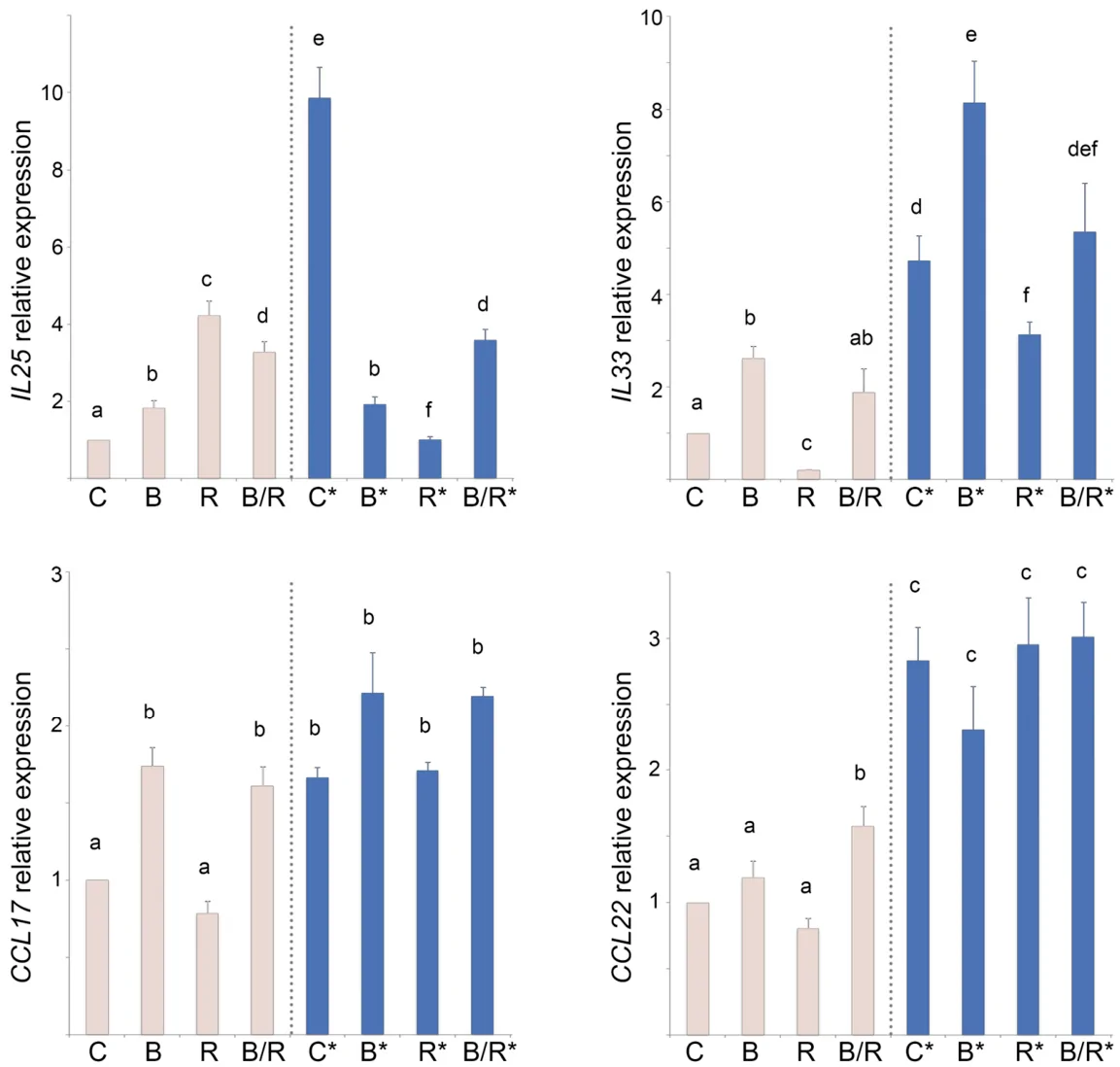
Cytokine gene expression in HaCaT cells. Gene expression analysis by real-time PCR in HaCaT cells, unstimulated and stimulated with IFN-γ at a final concentration of 20 ng/mL (technical triplicate representative of three biological replicates). Values were calculated using the 2^−ΔΔCt^ method and normalized to the unstimulated control. Standard deviation was calculated using the error propagation method. Letters above each bar indicate statistically different groups, as determined by multiple comparisons, with *p*-values < 0.05. *: stimulation with IFN-γ; C: Control; B: Bacteria, final concentration 0.2 × 10^9^; R: Resveratrol, final concentration 3 µM; B/R: Bacteria + Resveratrol.

In non-stimulated HaCaT cells, HI *L. reuteri*, resveratrol and their combination increased the expression of IL-25, while CCL17 expression was not modified by resveratrol but increased in the presence of HI *L. reuteri*. Also, IL-33 expression was significantly increased by HI *L. reuteri* but reduced by resveratrol. In summary, under non-induced conditions, HI *L. reuteri* increased the expression of IL-25, CCL17, and IL-33, whereas resveratrol increased IL-25 expression and reduced IL-33 expression. Under stress conditions, following activation, treatment with resveratrol, HI *L. reuteri*, and their combination reduced IL-25 expression (Figure 7). IL-33 expression was differentially modulated by HI *L. reuteri* and resveratrol. In particular, the presence of bacteria resulted in an increase in expression, while resveratrol produced a significant reduction.

HI *L. reuteri* induced significantly higher levels of IL-33 after stimulation compared to untreated and IFN-γ-stimulated cells. However, the combination of bacteria and resveratrol restored expression levels in untreated and stimulated cells similar to those of their controls. The stimulation resulted in overexpression of CCL17 and CCL22; meanwhile, the expression of CCL17 in unstimulated cells was increased by the presence of bacteria.

### 3.6. Cytokine and Epidermal Integrity Gene Expression in a 3D Reconstructed Human Skin Model (EpiDermFT^TM^)

Based on the results obtained in monolayer cell lines, the effects of resveratrol, HI *L. reuteri*, and their combination were further evaluated in a more physiologically relevant 3D reconstructed human skin model (EpiDermFT^TM^). IFN-γ was used as the inducing agent, and gene expression was monitored by real-time PCR.

Expression of cytokine-related genes (IL-25, IL-33, IL-37) was assessed 6 and 24 h after IFN-γ induction, while genes related to epidermal integrity, filaggrin (*FLG*), loricrin (*LOR*), and involucrin (*INV*), were evaluated after 24 h.

**Figure 8:**
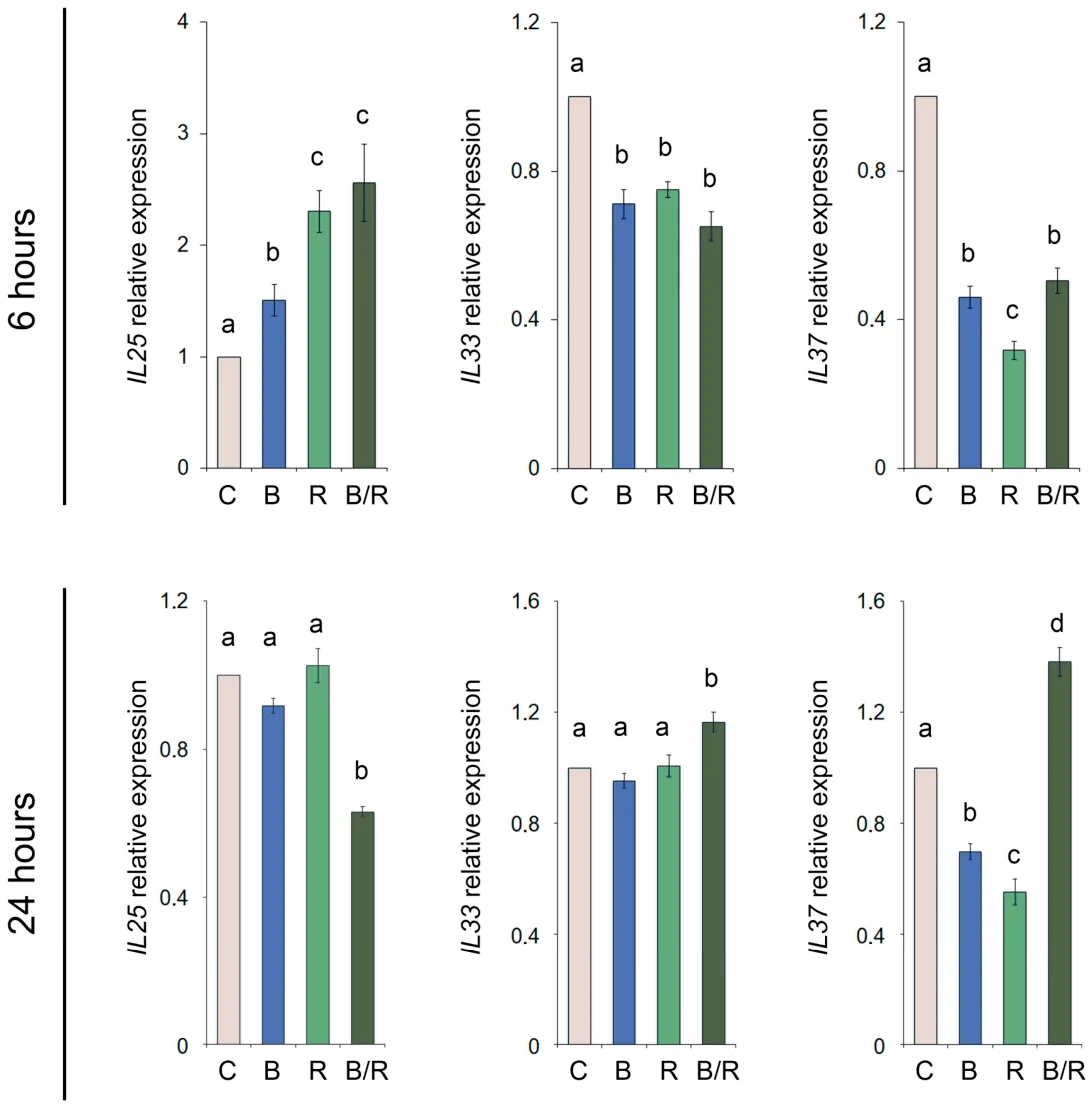
Cytokine gene expression in reconstructed 3D epithelia. Gene expression analysis by real-time PCR in reconstructed 3D epithelia stimulated with IFN-γ at a final concentration of 20 ng/mL (three technical triplicates). Values were calculated using the 2^−ΔΔCt^ method and normalized to the control. Standard deviation was calculated using the error propagation method. Letters above each bar indicate statistically different groups, as determined by multiple comparisons, with *p*-values < 0.05. C: Control; B: Bacteria, final concentration 0.2 × 10^9^; R: Resveratrol, final concentration 3 µM; B/R: Bacteria + Resveratrol.

Analysis revealed that resveratrol, HI *L. reuteri*, and their combination increased IL-25 transcription while reducing IL-33 and IL-37 expression after 6 h; meanwhile, at 24 h the combination of resveratrol and HI *L. reuteri* strongly reduced the expression of IL-25, similarly to what observed in IFN-γ stimulated HaCaT cells, left the expression of IL-33 almost unchanged, and on the contrary increased the levels of IL-37 compared to the controls (Figure 8).

**Figure 9:**
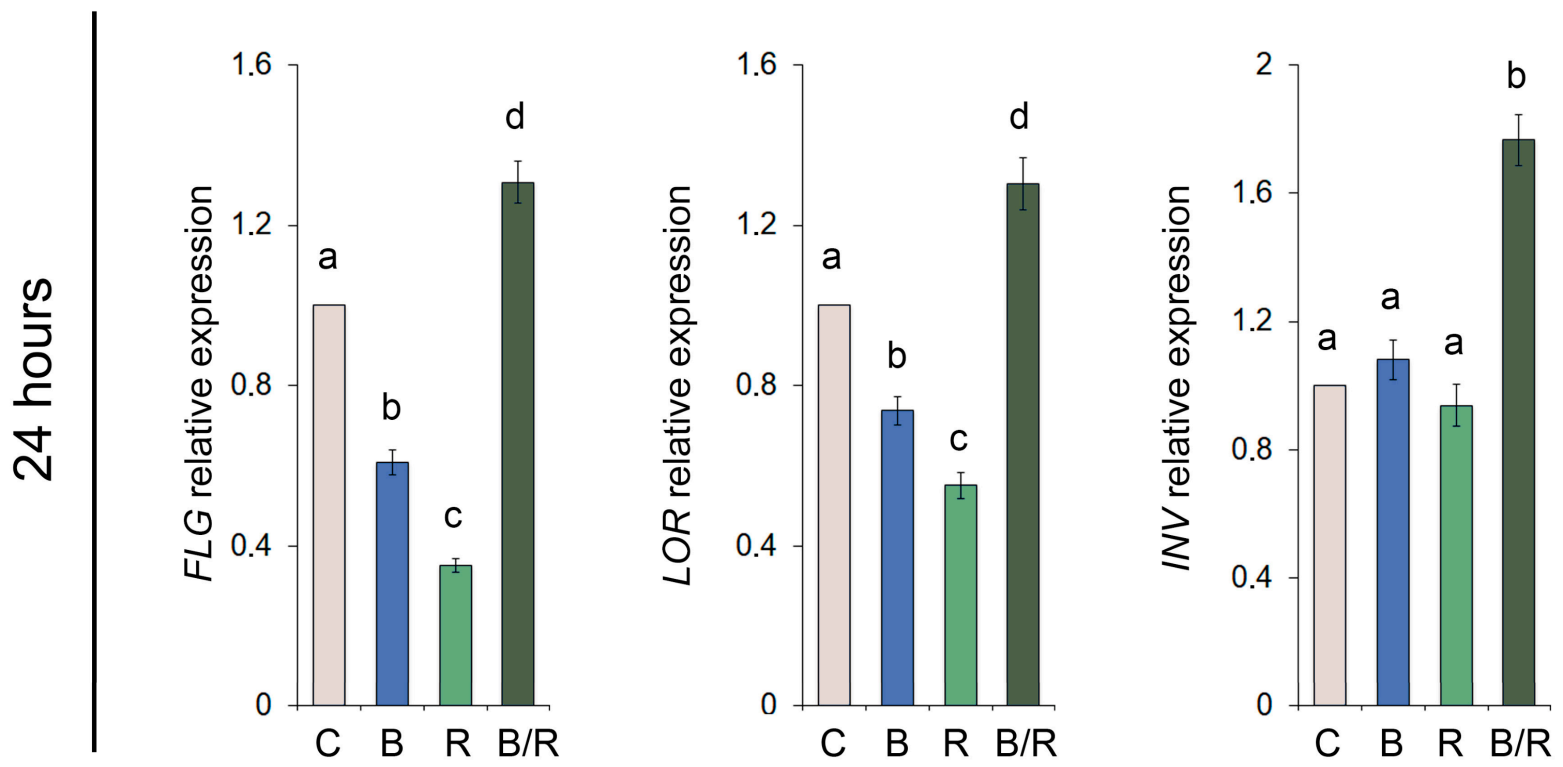
Epidermal integrity gene expression in reconstructed 3D epithelia. Gene expression analysis by real-time PCR in reconstructed 3D epithelia stimulated with IFN-γ at a final concentration of 20 ng/mL (three technical triplicates). Values were calculated using the 2^−ΔΔCt^ method and normalized to the control. Standard deviation was calculated using the error propagation method. Letters above each bar indicate statistically different groups, as determined by multiple comparisons, with *p*-values < 0.05. C: Control; B: Bacteria, final concentration 0.2 × 10^9^; R: Resveratrol, final concentration 3 µM; B/R: Bacteria + Resveratrol.

Regarding the expression of epidermal integrity genes, after 24 h treatment with resveratrol or HI *L. reuteri* alone significantly decreased *FLG* and *LOR* levels compared to controls, while *INV* remained unchanged. In contrast, combined treatment increased the expression of *FLG*, *LOR*, and *INV*, indicating a distinct result compared to single treatments (Figure 9) and a potential beneficial effect during infection.

## 4. Discussion

This study investigated the antibacterial effect of *Limosilactobacillus reuteri*, LMG P-27481, alone or in combination with resveratrol, against URT pathogens.

Resveratrol is an antiviral agent that has been shown to inhibit the replication of various respiratory viruses both in vitro and in vivo [31]. Frequently, a viral infection of the upper respiratory tract leads to a reduction in local defences, resulting in the development of bacterial infections by pathobionts, inherent members of the microbiota that turn harmful when the ecosystem is unbalanced. In the present study, we explored the efficacy of a probiotic strain combined with resveratrol in mitigating viral-induced damage and inhibiting the subsequent proliferation of bacterial pathogens.

### 4.1. Antibacterial Effect of L. reuteri SCS and Resveratrol

The assays were performed using spent culture supernatants (SCS) at two pH values (4.4 and 7) alone or in combination with resveratrol. Our results demonstrate that the tested strain of *L. reuteri* produces molecules able to inhibit the growth of respiratory pathobionts (*Staphylococcus aureus*, *Streptococcus pyogenes*, *Streptococcus pneumoniae*, *Moraxella catarrhalis*, *Haemophilus influenzae*). It is well established that bacteria belonging to the *Lactobacillus* genus produce lactic acid through carbohydrate fermentation; this process is a hallmark of their probiotic activity, as it effectively inhibits the growth of pathogens. The antibacterial effect observed in our experiments is, however, only partially due to the acidic pH produced by the lactic acid released. Indeed, after pH neutralization of SCS, a strong inhibitory activity towards Gram-negative bacteria (*M. catarrhalis*, *H. influenzae*) was still present, suggesting that other *L. reuteri* metabolites mediate the contact-independent inhibition of these respiratory pathobionts. Furthermore, culture medium adjusted to pH 4.5 did not affect the growth of *M. catarrhalis*, suggesting that the inhibitory effect observed against Gram-negative pathobionts is not solely pH-dependent.

The presence of a sub-inhibiting amount of resveratrol did not modify the antibacterial effect of SCS against Gram-positive bacteria, but was strongly effective on the multiplication of *M. catarrhalis* and *H. influenzae* at both acidic and neutral pH. The effects observed with the combination of *L. reuteri* SCS and resveratrol on Gram-negative bacteria are compelling and warrant further investigation to elucidate the underlying mechanisms of action.

Synergism between resveratrol and antimicrobial substances has been reported. Co-administration of subinhibitory concentrations of resveratrol increased the activity of antimicrobials against *S. aureus* and other Gram-positive pathogens and sensitized *S. aureus* towards human beta defensin 4-mediated killing [37]. Resveratrol inhibits bacterial ATP synthase function, and this activity has been linked to its effect on the increased efficacy of aminoglycosides and killing by human antimicrobial peptides [37].

We hypothesize that the enhanced SCS-mediated inhibition of Gram-negative bacteria observed in this study may originate from similar mechanisms or other cellular alterations induced by resveratrol. Indeed, resveratrol has been reported to exert various effects on bacteria, including DNA damage, impairment of cell division, and oxidative membrane damage. Furthermore, it inhibits metabolic enzymes, suppresses FtsZ gene expression, thereby disrupting Z-ring formation, and generates Cu (II)-peroxide complexes that lead to DNA cleavage and cell death. Additionally, resveratrol is known to inhibit the electron transport chain and F0-F1 ATPase, ultimately compromising cellular energy production [38].

### 4.2. Antibiofilm Effect

The antibacterial activity of *L. reuteri* LMG P-27481 SCS led to an investigation into whether its secreted metabolites could also interfere with biofilm dynamics, given the recognized role of biofilm in the persistence and recurrence of upper respiratory tract infections. Indeed, biofilms have been implicated in various respiratory infections, especially in children, including acute otitis media, adenoiditis, and protracted bacterial bronchitis, and are associated with recurrent and chronic conditions [39]. These results showed that SCS significantly reduced *S. pyogenes* LMG 21599 biofilm biomass in both disaggregation and prevention assays, whereas no statistically significant effect was observed for the other strains tested. The activity observed against *S. pyogenes* is consistent with the broader evidence that secreted metabolites from lactic acid bacteria can interfere with multiple stages of pathogen biofilm development through mechanisms including inhibition of quorum sensing, reduction in surface adhesion, and the antagonistic activity of organic acids and other secreted compounds [40].

### 4.3. Antioxidant Activity

A crucial requirement for probiotic strains intended for URT applications, which are typically anaerobic or microaerophilic, is the ability to tolerate the high oxygen concentrations characteristic of this environment.

The probiotic strain must be able to survive and remain metabolically active for at least a period sufficient to perform its health-protective activity. However, the antioxidant activities can also be exerted by the bacterial wall components and by the antioxidant products from bacterial metabolism. In our work, we therefore evaluated the antioxidant capacity of the strain under consideration using heat-inactivated *L. reuteri* to also avoid confounding elements as a result of the bactericidal reagents used in the tests.

In this study, we demonstrated the notable antioxidant capacity of the HI *L. reuteri* strain, both alone and in combination with resveratrol. Notably, across all employed assays (NBT, ABTS, and ORAC), the combination of resveratrol and HI *L. reuteri* demonstrated potent free radical scavenging activity, with variations in efficacy correlating to each specific assay.

Regarding the ORAC assay, the observed trend for resveratrol is consistent with the typical behaviour of redox-active polyphenols. These data suggest that while resveratrol acts as a potent ROS scavenger at low-to-intermediate concentrations, pro-oxidative effects may emerge at higher dosages. Interestingly, the inclusion of lactic acid bacteria (green curve) led to a decrease in the overall signal as resveratrol concentration increased. Given that these bacteria possess intrinsic ORAC activity, such a decline may point toward a coordinated pro-oxidant effect arising from the interaction between the polyphenols and the bacterial cells.

Overall, these experiments on redox activity confirmed the known ROS-scavenging ability of lactic bacteria [41] and resveratrol alone while demonstrating an intriguing additive effect that potentiates their antioxidant effect.

### 4.4. Cytokine Production and Gene Expression

We studied the effects of HI *L. reuteri*, resveratrol, and their combination on the production of IL-6, IL-8, TNF-α, and IL-1β in different cell types. As expected, HI *L. reuteri* exerted distinct effects across cell types. Specifically, in macrophages, the presence of bacteria led to a significant increase in IL-6, TNF-α, and IL-1β production, whereas in mucosal CaCo-2 cells, IL-8 was increased. Moreover, in keratinocytes, HaCaT HI *L. reuteri* determined a decline of IL-1β, which was similarly decreased by resveratrol.

These data indicate that HI *L. reuteri* does not merely suppress inflammation but rather acts as a context-dependent immunomodulator whose effects are shaped by both the cellular compartment and the inflammatory activation state.

Recent evidence further supports the concept that probiotic-mediated immunomodulation should not be interpreted as a simple anti-inflammatory phenomenon but rather as a dynamic process resulting from the bidirectional interaction between microbial-derived signals and host immune pathways. Selected probiotic strains have been shown to fine-tune innate immune responses through the activation of pattern-recognition receptors (PRRs) and the modulation of downstream signaling cascades, including NF-κB and MAPK pathways, thereby balancing antimicrobial defense with the preservation of epithelial homeostasis. Moreover, host–microbe interactions involve not only cytokine regulation but also reinforcement of barrier function and maintenance of immune tolerance, highlighting that transient immune activation may represent a physiological component of mucosal protection rather than a detrimental inflammatory response [42,43]. These emerging concepts are consistent with our findings, in which *L. reuteri* elicited distinct, cell-specific responses that collectively support effective host defense while limiting excessive tissue inflammation.

Accordingly, the increased production of IL-6, TNF-α and IL-1β observed in RAW264.7 macrophages should be interpreted as evidence of innate immune activation rather than a direct inflammatory effect. In macrophages, the robust induction of these cytokines, particularly following stimulation, is consistent with the concept of context-dependent immunomodulation and is compatible with the paradigm of “trained innate immunity” described for other beneficial bacterial strains. Through the engagement of PRRs, commensal bacteria can transiently activate macrophages, thereby enhancing early antimicrobial responsiveness, promoting neutrophil recruitment and pathogen clearance, while simultaneously preserving the capacity to engage regulatory pathways, including IL-10-mediated responses, that contribute to the restoration of immune homeostasis in anti-inflammatory settings [44].

Rather than representing opposing biological effects, the differential responses observed in macrophages and keratinocytes reflect their distinct physiological roles during host defence. While macrophages are specialized to initiate rapid antimicrobial responses, keratinocytes primarily function to preserve barrier integrity and limit excessive inflammatory signalling. Therefore, transient activation of innate immune responses in macrophages together with reduced IL-1β expression in keratinocytes represents complementary immunomodulatory mechanisms that optimize host defence while minimizing tissue damage and preserving skin homeostasis [45].

These results are in line with recent data reporting possible mechanisms of protection exerted by healthy upper respiratory microbiota through modulation of the immune environment. For instance, airway *Prevotella* was shown to enhance protection against *S. pneumoniae* by inducing TNF-α and neutrophil activation [24]. In the absence of known pathogens, Corynebacteria triggered a transient inflammatory response in both respiratory epithelial cells and murine models. This was characterized by the secretion of inflammatory mediators IL-8 and IL-6 from epithelial cells, alongside elevated levels of IL-6 and TNF-α in the nasal lavage of mice [46]. Collectively, our findings align with the emerging concept that upper respiratory tract commensals can enhance resistance to pathogens by transiently potentiating innate immune effector mechanisms, without necessarily promoting chronic inflammatory pathology. Importantly, these findings indicate that the transient increase in pro-inflammatory cytokines should not be interpreted as intrinsically detrimental, but rather as part of a tightly regulated innate immune response that enhances early pathogen clearance before the activation of resolution pathways and restoration of tissue homeostasis [47,48].

In intestinal epithelial cells, the preferential modulation of IL-8 relative to IL-6 and TNF-α following stimulation suggests that HI *L. reuteri* promotes a chemokine-oriented mucosal recruitment programme while selectively constraining inflammatory pathways associated with chronic epithelial injury [49,50]. Taken together with the macrophage data, these findings further support the concept that *L. reuteri* exerts cell type-specific immunomodulatory effects rather than uniformly suppressing inflammatory signalling across different tissues [42].

By contrast, keratinocytes, which represent a major epithelial source of the inflammasome-associated cytokine IL-1β, displayed reduced cytokine expression following exposure to both HI *L. reuteri* and resveratrol, suggesting a convergent epithelial-protective immunomodulatory effect [51]. Mechanistically, this effect may involve suppression of NF-κB-dependent inflammasome priming by the bacterial strain together with attenuation of oxidative stress-associated inflammatory signaling mediated by the polyphenol, ultimately limiting excessive local tissue injury and promoting epithelial immune homeostasis [52,53]. Although macrophage activation and attenuation of epithelial inflammatory responses may initially appear contradictory, these findings are consistent with the compartment-specific organization of mucosal immunity. Professional innate immune cells, such as macrophages, are responsible for initiating rapid antimicrobial responses following microbial recognition, whereas epithelial cells primarily function to preserve barrier integrity and minimize collateral tissue damage. Thus, transient activation of innate immune responses in macrophages, together with the regulation of inflammatory signalling in epithelial cells, represents complementary rather than opposing mechanisms that collectively promote effective host defence while maintaining tissue homeostasis [48,54].

Under stimulation, HI *L. reuteri* increased TNF-α and decreased IL-6 in mucosal CaCo-2 cells, whereas resveratrol increased IL-6. Moreover, HI *L. reuteri* induced significantly higher levels of TNF-α in mucosal Caco-2 cells after stimulation compared to untreated and stimulated cells. The contrasting effect of resveratrol, which increased IL-6 in Caco-2 cells, further underscores the complexity of polyphenol–microbiota interactions and suggests that the net output depends on the specific cell type, signalling context and concentration used.

Moreover, HI *L. reuteri*, resveratrol, and their combination may protect against the inflammatory response by reducing IL-6 production in Caco-2 cells and IL-25 expression in HaCaT cells. This potential competence is consistent with mounting evidence that selected probiotic strains can dampen type 2-biased cytokine responses while preserving or enhancing barrier-related functions [55].

The results from the 3D reconstructed human skin model (EpiDermFT^TM^) provided more physiologically relevant insights into the biological activity of resveratrol and HI *L. reuteri*, confirming and extending the preliminary observations in monolayer cell lines. The gene expression analysis of cytokines revealed a distinct temporal dynamic. After 6 h of IFN-γ treatment, the observed increase in IL-25 transcription, coupled with reductions in IL-33 and IL-37, suggests an immediate response to the inflammatory stimulus.

However, the most significant evidence emerged after 24 h, as the combined treatment with resveratrol and HI *L. reuteri* revealed a potent decrease in IL-25 expression, concomitant with the upregulation of IL-37 compared to control and single-treated samples. Since IL-37 is a fundamental anti-inflammatory cytokine that inhibits innate immunity [56], its upregulation suggests that the combined therapy actively promotes resolution of the inflammatory state induced by IFN-γ. The delayed induction of IL-37 by the combination, together with the reduction in IL- 25, indicates that HI *L. reuteri* and resveratrol cooperate to orchestrate a temporal switch from an early ‘alarm’ phase to a pro-resolving phase, in which broad suppression of innate immune signalling is coupled with dampening of alarmin-driven inflammation. This temporal reprogramming is compatible with the concept that IL-37 acts as a broad brake on innate responses [57], including macrophage and epithelial activation, and that its upregulation may limit collateral tissue damage during sustained inflammation exposure [58].

Moreover, the combined treatment on the EpiDermFT^TM^ model highlighted a particularly robust upregulation of skin barrier markers FLG, LOR, and INV expression at 24 h, with respect to the control and single treatments. Based on the observed recovery of structural markers and the established roles of AhR and SIRT1 in epidermal homeostasis [59,60], it is tempting to speculate that the combination of resveratrol and *L. reuteri* may partially act through these signalling pathways. However, this hypothesis requires direct experimental validation.

Although the epithelial models used in this study do not directly recapitulate the respiratory mucosa, the combined findings provide complementary mechanistic insight into conserved host defence pathways across barrier tissues. Specifically, the transient activation of macrophages supports an effective early innate immune response, whereas the coordinated modulation of alarmins (like IL-25) and anti-inflammatory cytokines (like IL-37) is critical for respiratory health. While IL-25 initiates localized type 2 immune responses to external insults [61], IL-37 actively dampens inflammation and supports tight-junction integrity [62], collectively preserving epithelial homeostasis and preventing chronic airway remodeling, providing a biologically plausible framework supporting the potential relevance of *L. reuteri* for respiratory mucosal defence. This interpretation is further supported by the emerging concept of the gut–lung axis, whereby modulation of epithelial immunity at barrier sites can influence immune responses within the respiratory tract [63]. Nevertheless, these observations remain mechanistic and require validation in dedicated respiratory epithelial models and in vivo studies.

### 4.5. Future Perspectives

In conclusion, this study demonstrated that *L. reuteri* LMG P-27481 exhibits antimicrobial activity against URT pathobionts. A combination of this strain of *L. reuteri* and resveratrol could have potential as a therapeutic or preventive strategy against URTI, in which these bacteria are involved. The results obtained in this work may have significant clinical implications. Viruses are the major cause of respiratory infections. Many respiratory viruses are inhibited by resveratrol. Reduced viral replication both decreases the intensity and duration of symptoms and reduces the local damage caused by viral multiplication, particularly on ciliary motility. This damage contributes significantly to bacterial superinfections. A product containing a combination of resveratrol and *L. reuteri* LMG P-27481 could represent an important weapon against most URTIs, reducing or delaying the administration of antibiotics and the consequent increase in antimicrobial resistance. Clearly, some limitations should be acknowledged, which prevent definitive conclusions from being drawn. These include: (i) lack of direct measurements of intracellular ROS levels; (ii) absence of both intracellular and extracellular metabolomic analyses; (iii) lack of detailed investigations into the specific molecular and cellular pathways involved; and (iv) low sample size in some experimental settings. Moreover, future preclinical and clinical investigations are required to substantiate the therapeutic potential of the combination of *L. reuteri* and resveratrol observed in this in vitro study.

## 5. Patents

The presented work was funded by NOOS S.r.l. (Rome, Italy) and involved the creation of a patent—ID: 102023000004017, ref. BI5778R-PDG-fp—together with its European extension (ref. BE2398R-PDG-fp), currently under evaluation.

## Figures and Tables

**Table 1:** MIC values of *L.reuteri* LMG P-27481 SCS and resveratrol on pathogens.

Strains	*L. reuteri* SCS (%), Native pH	*L. reuteri* SCS (%), Neutral pH	Resveratrol (µg/mL)
*S. aureus* NCTC 6571	50	>100	160
*S. pyogenes* LMG 21599	25	100	160
*S. pneumoniae* DSM 20566	25	100	80
*M. catarrhalis* DSM 9143	25	25	160
*H. influenzae* DSM 4690	12.5	12.5	80

MIC values are expressed as a percentage of SCS volume in the well.

**Table 2:** MIC values of *L. reuteri* LMG P-27481 SCS, at native and neutral pH, combined with 40 μg/mL of resveratrol.

Strains	*L. reuteri* SCS (%), Native pH + Resveratrol	*L. reuteri* SCS (%), Neutral pH + Resveratrol
*S. aureus* NCTC 6571	50	>100
*S. pyogenes* LMG 21599	25	100
*S. pneumoniae* DSM 20566	25	100
*M. catarrhalis* DSM 9143	1.56	3.12
*H. influenzae* DSM 4690	0.78	1.56

MIC values are expressed as a percentage of SCS volume in the well.

**Table 3:** Antioxidant activity of resveratrol, HI *L. reuteri* and their combination compared to ascorbic acid.

Free Radical Scavenging Activity	IC_50_			
	Ascorbic Acid (mM)	Resveratrol (mM)	Bacteria (cfu/mL)	Resveratrol + Bacteria (mM)
NBT Assay (^●^O_2_^−^)	0.665 ± 0.026	0.090 ± 0.012 **	2.6 × 10^7^ ± 1.5 × 10^6^	0.006 ± 0.002 ^#^ (2)
ABTS Assay (ABTS^●+^)	0.007 ± 2.39 × 10^−5^	0.010 ± 0.004	n.d.	0.003 ± 0.0007 (1)
Oxygen radical absorbance capacity	ORAC value (mM Trolox equivalents)			
ORAC Assay (R-O-O^●^)		27.397 ± 0.503 10 µM	42.753 ± 1.642 4.0 × 10^8^	18.269 ± 0.461 ^§§^ 10 µM + 4.0 × 10^8^

** *p*-value < 0.01 vs. ascorbic acid. ^#^ *p*-value < 0.05 vs. resveratrol. ^§§^ *p*-value < 0.01 vs. resveratrol. n.d.: not detected. (1) Combination prepared with a bacterial concentration of 8.0 × 10^7^ cfu/mL. (2) Combination prepared with a bacterial concentration of 2.0 × 10^7^ cfu/mL.

## Data Availability

The original contributions presented in this study are included in the article. Further inquiries can be directed to the corresponding authors.

## Abbreviations

The following abbreviations are used in this manuscript:

ABTS•^+^	2,2′-azinobis-(3-ethylbenzothiazoline-6-sulfonic acid) radical cation
BHI	Brain Heart Infusion
DMSO	Dimethyl sulphoxide
ELISA	Enzyme-Linked Immunosorbent Assay
MIC	Minimal Inhibitory Concentration
MTT	3-(4,5-dimethylthiazol-2-yl)-2,5-diphenyltetrazolium bromide
NBT	nitroblue tetrazolium
OD	Optical Density
ORAC	Oxygen Radical Absorbance Capacity
qPCR	quantitative polymerase chain reaction
SCS	Spent culture supernatant
URT	upper respiratory tract
URTI	upper respiratory tract infection
